# Snake Venom: Toxicology and Associated Countermeasures

**DOI:** 10.3390/toxins17050237

**Published:** 2025-05-10

**Authors:** Nicholas J. Youngman

**Affiliations:** Attexo, Level 4/315 Brunswick St, Fortitude Valley, QLD 4006, Australia; nickjyoungman@outlook.com

## 1. Special Issue Introduction

This Special Issue aims to provide insight into the understudied toxicological effects induced by snakebite envenoming, as well as to highlight current and future countermeasures for reducing the extreme morbidity and mortality associated with this globally neglected tropical disease. It has been nearly a decade since the World Health Organization (WHO) designated snakebites as a neglected tropical disease in 2017, prompting a significant increase in research dedicated to snakebite envenoming. Notably, there has been substantial progress in our understanding of antivenom cross-reactivity, along with advances in the development and efficacy of novel alternative envenomation therapeutics. However, despite this surge in research, the venom activity of the vast majority of snake species remains either completely unknown or poorly understood. In addition, estimates of mortality and morbidity continue to rise, with approximately 125,000 fatalities and up to 5.5 million people affected annually. As the global human population continues to grow, particularly in socio-economically vulnerable regions where human–animal conflict is increasing and encroachment into snake habitats is ever present, these numbers could continue to rise without proper intervention.

The complex nature of snake venom—characterized by a multitude of toxin types and variations—poses a unique challenge for researchers, as the range of biological activities and effects can vary significantly between species, populations, individuals, and even testing methodologies. Even species considered as well studied in both clinical and experimental settings still exhibit previously undocumented venom activities. A deeper understanding of venom variation is proving to be both advantageous and limiting. While antivenom cross-reactivity has shown efficacy across some clades with broad geographical distribution, it has also been found ineffective for different populations of the same species. The overall positive impact of antivenom cannot be overstated; however, its limitations must also be acknowledged. Requirements such as cold storage, administration by trained medical professionals, varying degrees of specificity, and supply shortages in remote regions continue to hinder the success of antivenom treatments globally. This underscores the urgent need for broader, more easily accessible treatments is paramount.

All the studies featured in this Special Issue build upon the recent advancements in the field of snake venom research, contributing to the development of new strategies to mitigate the impact of this tropical disease.

## 2. An Overview of Published Articles

The immunoreactivity and in vivo efficacy studies conducted by Ariadna Rodriguez-Vargas et al. (Contribution #1) from the Instituto Nacional de Salud (INS) of Colombia on the cross-reactivity of polyvalent anticoral antivenom against the heterologous endemic venoms of *Micrurus medemi*, *M. sangilensis*, and *M. helleri* form a critical component of the foundational work required to immediately reduce mortality and morbidity associated with snakebite envenoming [1]. Coral snakes (*Micrurus*) comprise a diverse, highly venomous, and often difficult-to-identify clade across Central and South America; thus, identifying broadly applicable treatment options is essential. This study provides important baseline data on cross-reactivity and establishes in vivo pre-clinical efficacy for the use of an established antivenom against a mixture of venoms not used in the original immunizing process. The INS anticoral antivenom showed significant neutralization of *M. medemi* and *M. sangilensis* venoms in mice, corroborating the immunoreactivity assay results. It also showed noticeable cross-reactivity and in vivo neutralization towards *M. helleri* venom. However, the authors recommend evaluating the suitability of alternative antivenom treatment options for *M. helleri* or incorporating its venom into the immunizing mixture to enhance therapeutic efficacy.

Ryoichi Shirai et al. (Contribution #2) investigated the phospholipase A_2_ (PLA_2_) inhibitory properties of snake sera leucine-rich α_2_ glycoproteins (LRGs) [2]. Evolution has led to a variety of adaptations within the animal kingdom, enabling certain species to develop either immunity or resistance to snakebite envenoming. Many venomous snakes themselves possess such adaptations, offering resistance against envenomation by predators, prey, or conspecifics. In this study, PLA_2_ inhibitory proteins were extracted from the sera of various venomous snake species, with LRGs purified from *Elaphe climacophora, Gloydius brevicauda, Laticauda semifasciata, Naja kaouthia*, and *Protobothrops flavoviridis*. Binding analyses and inhibition enzymatic assays revealed varying levels of PLA_2_ activity inhibition induced by the purified LRGs. These findings highlight the role of LRGs as a defense mechanism and suggest their potential in the development of novel therapeutics, making them a compelling subject for further detailed investigation [2].

The search for novel therapeutics against snakebite envenoming extends beyond snakes themselves, with potential therapeutic compounds being discovered across diverse sources in the natural world. Camila Castro-Pinheiro et al. (Contribution #3) demonstrated that two fucoidan sulfated polysaccharides, extracted from brown seaweed (*Fucus vesiculosus* and *Undaria pinnatifida*), show significant therapeutic potential [3]. The study assessed the ability of these polysaccharides to suppress the coagulotoxicity associated with *Bothrops* envenoming. The ongoing biodiscovery of novel therapeutic options, such as those presented by Castro-Pinheiro et al., is both promising and essential. There is a critical need for a broad-spectrum, multi-target, first aid therapeutic that can be easily administered in the field immediately after envenomation. Due to the complex and cascading effects of coagulotoxins—often resulting in severe systemic and local damage—immediate neutralization is vital. Notably, fucoidan sulfated polysaccharides remain a distinct area of interest in antivenom research.

Zichen Qiao et al. (Contribution #4) investigated the eastern long-nosed viper (*Vipera ammodytes meridionalis*), contributing valuable insights into antivenom efficacy. This species was found to exhibit an ontogenetic shift in procoagulant potency, with all three tested antivenoms proving effective against both neonate and adult venoms [4]. Significant ontogenetic changes in venom potency and activity are becoming more frequently reported in various species, especially as neonate venom has become available due to increased captive breeding programs. In this case, for the eastern long-nosed viper, the shift does not pose a clinical concern; however, future studies should endeavor to assess this phenomenon for other species not yet thoroughly examined.

Mimi Lay and Wayne Hodgson (Contribution #5) evaluated the venom of one of the most medically important snake genera, *Daboia* spp. (Russell’s vipers), which are geographically widespread across Asia and responsible for a significant proportion of snakebites cases within these regions. In vitro assays identified the key components of the venom from Thai and Javanese *Daboia siamensis* that are primarily responsible for neurotoxic activity. The researchers also assessed the effectiveness of varespladib in inhibiting neurotoxicity [5], comparing its efficacy to that of the currently available Thai *D. siamensis* monovalent antivenom [5].

Sudharshan Rao et al. (Contribution #6) present a comprehensive review of the role snake venom proteins play in post-envenomation inflammation [6]. The review covers both the mechanisms underlying toxin activity associated with the dominant protein families such as PLA_2_, metalloproteinases, serine proteases, C-type lectins, cysteine-rich secretory proteins, and L-amino acid oxidases, as well as the current treatment strategies [6]. A prominent challenge in treatment is timing, with treatments often being delayed. Even when an antivenom is administered before systemic effects develop, the local effects of the toxins and the cascading inflammatory response have often already begun. This is compounded by the delayed onset of most anti-inflammatory drugs used during treatment. This review provides a broad overview of current therapeutic approaches and offers insights into more recent advancements and emerging research within the field.

The final contribution to this Special Issue, authored by Eduardo M.G. Fernández et al. (Contribution #7), focuses on the venom activity of the medically significant *Bothrops* genus. The study collected valuable data on the long-term effects of *Bothrops* envenoming in the Manaus region of the western Brazilian Amazon [7]. Fifty participants over 18 years of age were assessed three to twelve months post-envenomation, following the guidelines of the World Health Organization Disability Assessment Schedule 2.0. Patients reported a range of neuromusculoskeletal and sensory disabilities, with 29 patients displaying mild to extreme impairment. These included, but were not limited to, difficulties in walking or moving the affected limb, chronic pain, and reduced strength and range of joint motion [7]. In socioeconomically vulnerable regions, such as the Brazilian Amazon, long-term disabilities from snakebites threaten the livelihoods of individuals who depend on physical wellbeing for their livelihoods. Studies such as this are essential in capturing the full scope of snakebite morbidity. Detailed long-term studies remain limited, making this study particularly valuable for this heavily impacted region. The findings of this study also reinforce the critical need for first-aid treatments; the majority of participants in the study suffered from a long delay in receiving antivenom treatment and initially presented with severe local effects, bacterial infections, necrosis, and compartment syndrome. Many of these musculoskeletal complications can only be mitigated with immediate therapeutic treatment, and each moment of delayed treatment leads to further deterioration.

## 3. Conclusions

The studies published in this Special Issue continue to deepen our understanding of snakebite envenomation, current therapeutic treatments, and novel treatment options that hold promise for the future. Venomous snakes present a stark dichotomy in human society; they are both feared as animals that have the potential to cause death and lifelong disability, yet they are also vital components of their ecosystems and loved by many for their fascinating and beautiful nature. Coexistence with venomous snakes is crucial, and it is only possible through continued and increased global effort to further elucidate every aspect of their venom from all perspectives. Assumptions about antivenom cross-reactivity, venom potency, and venom activities have historically led to an unknown, but undoubtedly significant, proportion of mortality and morbidity. Thus, the ongoing efforts of researchers worldwide is paramount to ensure a comprehensive understanding of venom activities and to optimize global treatment strategies.

## Data Availability

Not applicable.
